# Strengthening Health Systems to Provide Rehabilitation Services

**DOI:** 10.1097/PHM.0000000000000753

**Published:** 2017-05-19

**Authors:** Etienne Krug, Alarcos Cieza

**Affiliations:** From the Department for Management of Noncommunicable Diseases, Disability, Violence and Injury Prevention, World Health Organization, Geneva, Switzerland.

The world faces new challenges in light of health and demographic trends, populations are ageing, and the number of people living with noncommunicable diseases and the consequences of injuries is increasing.^1–3^ The health, social, and economic consequences of these trends should serve as a call to policy makers to invest not only in health services that reduce mortality and morbidity but also in those that improve functioning and consequently well-being. These latter outcomes are at the core of rehabilitation, yet rehabilitation services are often underdeveloped, underresourced, and undervalued.

A dramatic increase in the absolute number of years lived with disability combined with a rising prevalence of severely disabling conditions has led to a demand for rehabilitation that is largely going unmet. Seventy-four percent of years lived with disability are the result of health conditions for which rehabilitation may be beneficial.^4^ The prevalence of health conditions associated with severe levels of disability has increased by nearly 23% since 2005.^5^ However, in many parts of the world, the size of the rehabilitation workforce is insufficient. For example, in the World Health Organization African and Eastern Mediterranean Regions, the number of trained professionals required to meet the demand for rehabilitation services (such as occupational therapists, physiotherapists, and speech therapists) is estimated to be a tenth of that required.^4^

Factors contributing to the unmet need for rehabilitation services include poor accessibility, transport barriers, high out-of-pocket expenses, and long waiting times.^6,7^ An additional factor is a lack of awareness of the need for rehabilitation—what it is, what it does, and whom it may benefit. Rehabilitation comprises a set of interventions designed to reduce disability and to optimize functioning in individuals with health conditions in interaction with their environment. As such, it is not restricted to a minority group of persons with disabilities or those with significant long-term impairments. Rehabilitation is also relevant to people experiencing limitations in functioning associated with ageing, an injury, or other conditions. For example, evidence indicates that rehabilitation improves functioning for those who have had acute myocardial infarction or strokes, while it can also have positive results for those with musculoskeletal or mental health conditions.^8–11^

The role that rehabilitation plays in maximizing the impact of other health services—such as surgical interventions, trauma care, and management of noncommunicable diseases—and its potential for significant cost savings are also frequently misunderstood and underestimated.^12^ For example, rehabilitation has been found to be beneficial in reducing length of stay in hospitals and decreasing readmissions, thus mitigating the negative social and health risks associated with prolonged hospitalizations. In the context of complex conditions that require intensive and highly specialized rehabilitation, cost savings to both the individual and the health sector may be realized. By improving a person's ability to participate more fully in everyday life, rehabilitation reduces the costs related to ongoing care and support and may accelerate the ability to return to education or employment.

Rehabilitation is part of universal health coverage and should be incorporated into the package of essential services, along with prevention, promotion, treatment, and palliation. To this end, on February 7, 2017, World Health Organization, Member States, international and professional organizations, nongovernmental organizations. and rehabilitation experts issued “Rehabilitation 2030: a call for action,” a commitment to key actions to strengthen rehabilitation services in Member States.^4^ These actions include the following: improving rehabilitation governance and investment, expanding a high-quality rehabilitation workforce, and enhancing rehabilitation data collection. The commitment to strengthen health systems to provide rehabilitation services should make it possible for millions of people not only to live longer but also to live well.

## References

[1] World Health Statistics 2016 Monitoring Health for the SDGs. Geneva: World Health Organization; 2016

[2] GBD 2015 Disease and Injury Incidence and Prevalence Collaborators. Global, regional, and national incidence, prevalence, and years lived with disability for 310 diseases and injuries, 1990–2015: a systematic analysis for the Global Burden of Disease Study 2015. *Lancet* 2016;388:1545–602 27733282 10.1016/S0140-6736(16)31678-6 PMC5055577

[3] Chatterji S Byles J Cutler D: Health, functioning, and disability in older adults–present status and future implications. *Lancet* 2015;385:563–75 25468158 10.1016/S0140-6736(14)61462-8 PMC4882096

[4] Rehabilitation 2030: A Call for Action. Geneva: World Health Organization; 2017 Available at: http://www.who.int/disabilities/care/ConceptNote.pdf?ua=1. Accessed February 8, 2017

[5] The Need to Scale up Rehabilitation. Geneva: World Health Organization; 2017 Available at: http://www.who.int/disabilities/care/NeedToScaleUpRehab.pdf?ua=1. Accessed February 8, 2017

[6] Eldar R Kullmann L Marincek C: Rehabilitation medicine in countries of central/eastern Europe. *Disabil Rehabil* 2008;30:134–41 17852214 10.1080/09638280701191776

[7] Bjarnason-Wehrens B McGee H Zwisler AD: Cardiac rehabilitation in Europe: results from the European cardiac rehabilitation inventory survey. *Eur J Cardiovasc Prev Rehabil* 2010;17:410–8 20300001 10.1097/HJR.0b013e328334f42d

[8] Dalal HM Doherty P Taylor RS: Cardiac rehabilitation. *BMJ* 2015;351:h5000 26419744 10.1136/bmj.h5000 PMC4586722

[9] Pollock A Baer G Campbell P: Physical rehabilitation approaches for the recovery of function and mobility after stroke. *Stroke* 2014;45:e202 10.1002/14651858.CD001920.pub3 PMC6465059 24756870

[10] Williams RM Westmorland MG Lin CA: Effectiveness of workplace rehabilitation interventions in the treatment of work-related low back pain: a systematic review. *Disabil Rehabil* 2007;29:607–24 17453982 10.1080/09638280600841513

[11] Crowther R Marshall M Bond G: Vocational rehabilitation for people with severe mental illness. *Cochrane Database Syst Rev* 2001:D003080 10.1002/14651858.CD003080 PMC4170889 11406069

[12] Howard-Wilsher S Irvine L Fan H: Systematic overview of economic evaluations of health-related rehabilitation. *Disabil Health J* 2016;9:11–25 26440556 10.1016/j.dhjo.2015.08.009

